# A Novel Conductometric Urea Biosensor with Improved Analytical Characteristic Based on Recombinant Urease Adsorbed on Nanoparticle of Silicalite

**DOI:** 10.1186/s11671-016-1310-3

**Published:** 2016-02-25

**Authors:** T. P. Velychko, О. О. Soldatkin, V. G. Melnyk, S. V. Marchenko, S. K. Kirdeciler, B. Akata, A. P. Soldatkin, A. V. El’skaya, S. V. Dzyadevych

**Affiliations:** Institute of Molecular Biology and Genetics of NAS of Ukraine, Zabolotnogo Street 150, 03143 Kyiv, Ukraine; Taras Shevchenko National University of Kyiv, Volodymyrska Street 64, 01003 Kyiv, Ukraine; Department of Electrical and Magnetic Measurements, Institute of Electrodynamics of National Academy of Sciences of Ukraine, 56, Peremohy Ave., Kyiv-57, 03680 Ukraine; Micro and Nanotechnology Department, Middle East Technical University, Ankara, 06531 Turkey; Central Laboratory, Middle East Technical University, Ankara, 06531 Turkey

**Keywords:** Silicalite, Enzyme, Biosensor, Recombinant urease, Conductometry

## Abstract

Development of a conductometric biosensor for the urea detection has been reported. It was created using a non-typical method of the recombinant urease immobilization via adsorption on nanoporous particles of silicalite. It should be noted that this biosensor has a number of advantages, such as simple and fast performance, the absence of toxic compounds during biosensor preparation, and high reproducibility (RSD = 5.1 %). The linear range of urea determination by using the biosensor was 0.05–15 mM, and a lower limit of urea detection was 20 μM. The bioselective element was found to be stable for 19 days. The characteristics of recombinant urease-based biomembranes, such as dependence of responses on the protein and ion concentrations, were investigated. It is shown that the developed biosensor can be successfully used for the urea analysis during renal dialysis.

## Background

Urea [(NH_2_)_2_CO] is synthesized in the liver and is the final product of detoxification of endogenous ammonia, which is formed due to the decay of proteins and other nitrogen-containing compounds. The synthesized urea is released from the liver into the blood and transported to the kidneys where it is filtered and excreted with the urine. Normally, the urea concentration in humans ranges from 2.5 to 7.5 mM [1], but the rate of its synthesis, and thus the concentration, increase partially if either the protein-rich food is used, or endogenous catabolism is enhanced under the conditions of starvation, or the tissues are damaged, etc. However, a drastically elevated level of urea (50–150 mM) in the blood plasma indicates a kidney dysfunction. Such abnormal level of urea may be reduced to 10 mM by hemodialysis or peritoneal dialysis [2]. Therefore, determination of the urea concentration is of vital importance in biomedical and clinical assays. To this end, numerous methods are developed including gas chromatography [3], spectrophotometry [4, 5], and fluorometry [6]. The disadvantages of the above methods are dependence of the results on the sample pretreatment, long-time procedure, the need for highly qualified personnel, and impossibility of online measurements.

An alternative to the above methods is the use of biosensors—miniature analytical devices without the drawbacks listed. Numerous biosensors have been developed to date for urea analysis in biological samples including potentiometric [7–9], conductometric [10–12], and amperometric [13–15]. However, all of them have two significant disadvantages. First, they have rather a narrow linear range of determination and it is a characteristic trait of urease-based biosensors, which are used in urea assays. To solve this challenge, earlier, we have proposed recombinant urease from *E. coli* with high Km to shift the linear range to higher urea concentrations [16]. Another drawback of the known urea biosensors is associated with the immobilization of biological material on the surface of transducers. Urease can be immobilized by covalent binding [17], physical adsorption [18], binding with polymers [14, 19, 20], or coupling to the transducer surface [21, 22]. Some problems are intrinsic for these methods. They are as follows: the loss of enzyme activity, unstable reproducibility of biosensor signals, and toxicity of the compounds, which induce the binding. The latter is a particular problem in the determination of the enzyme activity in biological samples. To overcome these difficulties, zeolites were proposed as carriers for enzyme adsorption. The zeolites are slightly toxic and highly resistant to mechanical, chemical, and thermal injuries [23]; therefore, the zeolite-based biosensors can be used for multicomponent biological samples. This method of immobilization demonstrated promising results in a number of enzyme biosensors [24–26].

To create the biosensor for urea determination in biological samples, it was necessary to address the described problems simultaneously. This study was aimed at the development of the biosensor for highly accurate and stable determination of urea in a wide range of concentrations. For the purpose, it was proposed to use recombinant urease adsorbed on the surface of zeolite-modified conductometric transducers.

## Methods

### Materials

The enzyme urease (EC 3.5.1.5) from *E. coli* was used in the work, activity 150 U/mg, produced from “USBiological” (USA). Bovine serum albumin (BSA, fraction V) and urea were obtained from “Sigma-Aldrich Chemie” (Germany). Working buffer was phosphate buffer (KH_2_P0_4_-Na0H), pH 7.4, from “Helicon” (Moscow, Russia). Other inorganic compounds used were of analytical reagent grade.

Silicalite was synthesized in the Middle-East Technical University (Ankara, Turkey). To synthesize the silicalite crystals, the gel 1TPAOH:4TEOS:350 H_2_O was prepared. To obtain the formula, tetraethoxysilane (TEOS) and tetrapropylammonium hydroxide (TPAOH) were mixed with distilled water under constant stirring for 6 h at room temperature. The crystallization took place at 125 °C for 18 h. The resulting solid material was washed four times with distilled water under centrifugation. The products were dried at 100 °C overnight. The size of silicalite particles was approximately 250 nm.

### Conductometric Transducers

The conductometric transducers used in the work were produced at V.Ye. Lashkarev Institute of Semiconductor Physics, NASU (Kyiv, Ukraine) in accordance with our recommendations. They were 5 × 30 mm in size and consisted of two pairs of identical gold interdigitated electrodes on the sital substrate. The transducer design, preparation, and application are presented in detail in [27].

### Scheme of Experimental Setup for Conductometric Measurements

**Fig. 1:**
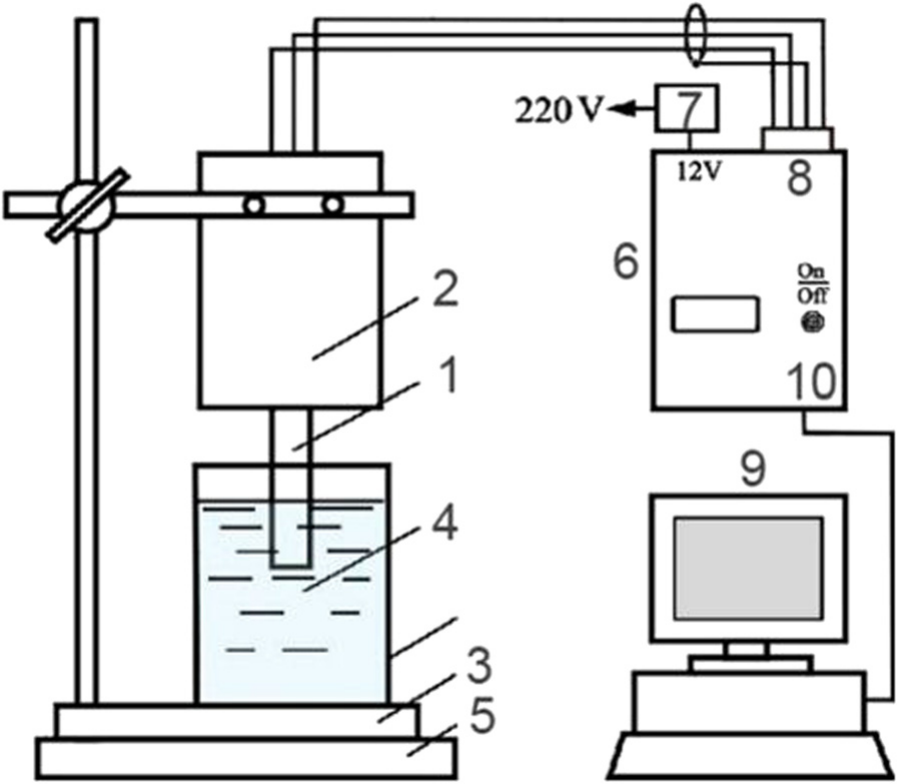
Scheme of connection of MXP-3 device in a system for conductometric measurements

The portable conductometry МХР-3, developed and manufactured at the Institute of Electrodynamics, NASU (Fig. 1) served as a measuring device. The sensor block consisted of differential conductometric transducer (1), holder for conductometry (2), and support (3). Under measurements, the working cell (4) with test solutions is placed on the support, and the whole sensor block is set on the magnetic stirrer (5).

The portable device MXP-3 (6) is connected to the electrical supply network via adapter (7), to the sensor block—with wires via contact (8), and to the personal computer (9) with a suite of related software—via contact (10). The current of frequency 37 kHz and amplitude 14 mV was used. First, conductometric transducer (1) was connected to the holder (2) and an initial baseline was obtained. Then, the tested substance was added to the working cell. The responses were recorded on a personal computer screen.

### Preparation of Bioselective Elements

The procedure of urease adsorption on silicalite was developed earlier [28]. The transducers previously coated with silicalite were used; 0.15 μl of 5 % recombinant urease solution in 20 mM phosphate buffer, рН 6.5, was deposited onto one pair of electrodes and 0.15 μl of 5 % BSA in the analogous buffer—onto the other (reference) pair. Afterwards, the transducers were exposed to complete air-drying (for 17 min). Neither glutaraldehyde nor any other auxiliary compounds were used. Next, the transducers were submerged into the working buffer for 20 to 30 min to wash off the unbound enzyme. After experiments, the transducer surface was cleaned from silicalite and adsorbed urease with ethanol-wetted cotton.

### Procedure of Measurement

The measurements were carried out in 5 mM phosphate buffer, pH 6.5, at room temperature in an open cell with constant stirring. The necessary substrate concentration in the working cell was obtained by addition of aliquots of the substrate stock solutions. All experiments were conducted in four series. The non-specific changes in output signal associated with the fluctuations in temperature, environmental pH, and electrical noise were avoided due to the differential mode of measurements.

## Results and Discussion

### Characterization of Silicalite

**Fig. 2:**
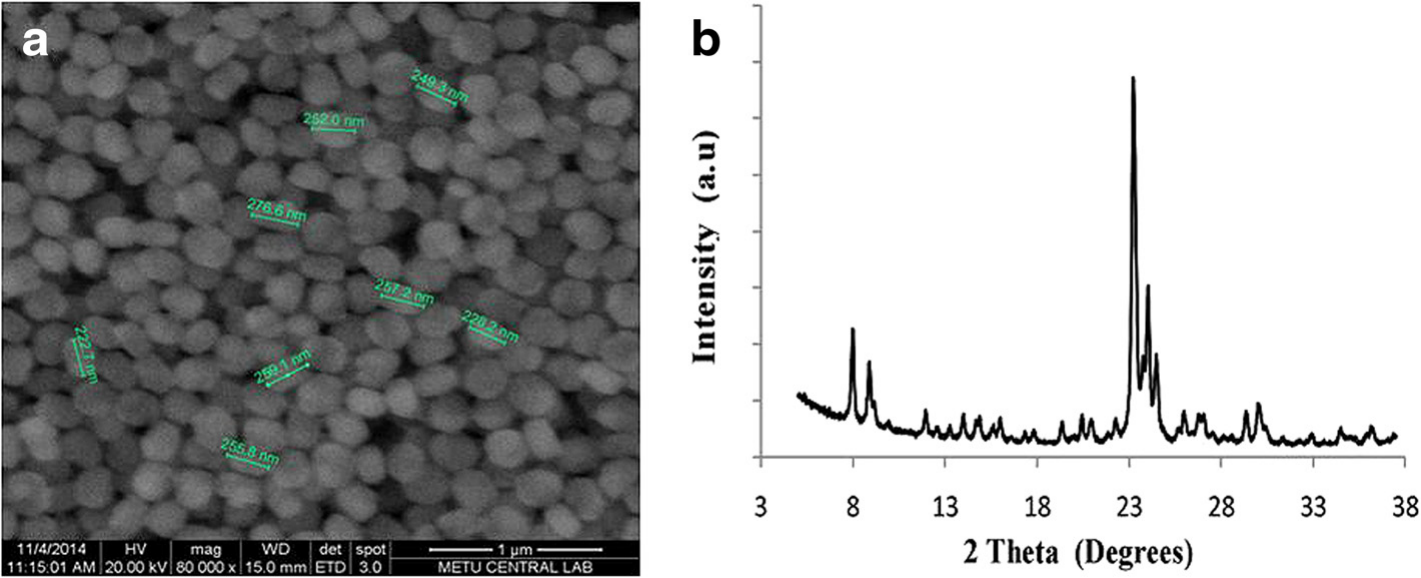
Scanning electron microscopy image of KK46 silicalite (**a**) and XRD spectrum of KK46 silicalite (**b**)

The resulting samples were characterized by powder X-ray diffraction (XRD) using Ni-filtered Cu-Kα radiation in a Philips PW 1729. Scanning electron microscopy (SEM) analysis were performed in a 400 Quant FEI. The surface area of the samples was obtained by multipoint BET, whereas the pore size and pore volumes were obtained by Saito-Foley (SF) and *t*-plot methods. The method of sample preparation included their outgassing under vacuum at 300 K for 4 h before analysis. The morphologies of the produced silicalite can be seen in Fig. 2a.

According to the X-ray diffraction data, presented in Fig. 2b, all samples exhibited the characteristic diffraction lines of their structures. In Table 1, particle sizes, pore sizes, surface area, and pore volume are given.

**Table 1:** Characteristics of KK46 silicalite

Particle size	Surface area	Pore volume	Pore size
≈250 nm	296 m^2^/g	0.12 cc/g	0.42 nm
SEM	Multipoint BET	S-F method	S-F method

### Analytical Characteristics of Biosensor

The biosensor operation is underlain by the enzymatic reaction, which takes place in the membrane containing recombinant urease deposited on the surface of conductometric transducer:

$$ \begin{array}{c}\mathrm{Recombinant}\ \mathrm{urease}\\ {}\mathrm{Urea} + 2{\mathrm{H}}_2\mathrm{O} + {\mathrm{H}}^{+}\to\ {{2\mathrm{N}\mathrm{H}}_4}^{+}{{ + \mathrm{H}\mathrm{C}\mathrm{O}}_3}^{\hbox{-}}\end{array} $$

In the course of enzymatic reaction, the local concentration of ions in the enzyme membrane increases. This changes the solution conductivity, which is registered by the conductometric transducer [29]. These changes and, consequently, biosensor responses are proportional to the concentration of urea.

### Effect of Solution Parameters on Value of Biosensor Response

As known, the conductometric method is based on the measuring of changes in the sample solution conductivity. This change in conductivity may depend on both the enzymatic reaction itself and the characteristics of solution in which this reaction occurs. So, first, an influence of the solution parameters (ionic strength, buffer capacity, protein concentration in the solution) on the value of sensor response was studied.

**Fig. 3:**
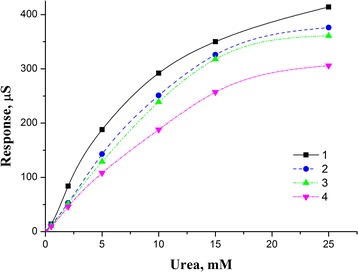
Dependence of value of biosensor response to urea concentration on various concentrations of buffer solution (*1* 2.5 mM, *2* 5 mM, *3* 10 mM, *4* 25 mM). Measurements were carried out in phosphate buffer, pH 7.35

The buffer capacity of human blood is relatively high due to the presence of proteins and buffer salts. To avoid the effects of blood buffer capacity, the concentration of working buffer in the measurement cell should therefore be not less than 5 mM. The dependence of biosensor responses on the urea concentration at various concentrations of buffer solution (2.5, 5, 10, 25 mM) is shown in Fig. 3. As seen, with increasing buffer capacity, the biosensor responses to urea decrease, but are still good enough for concentration 5 mM.

**Fig. 4:**
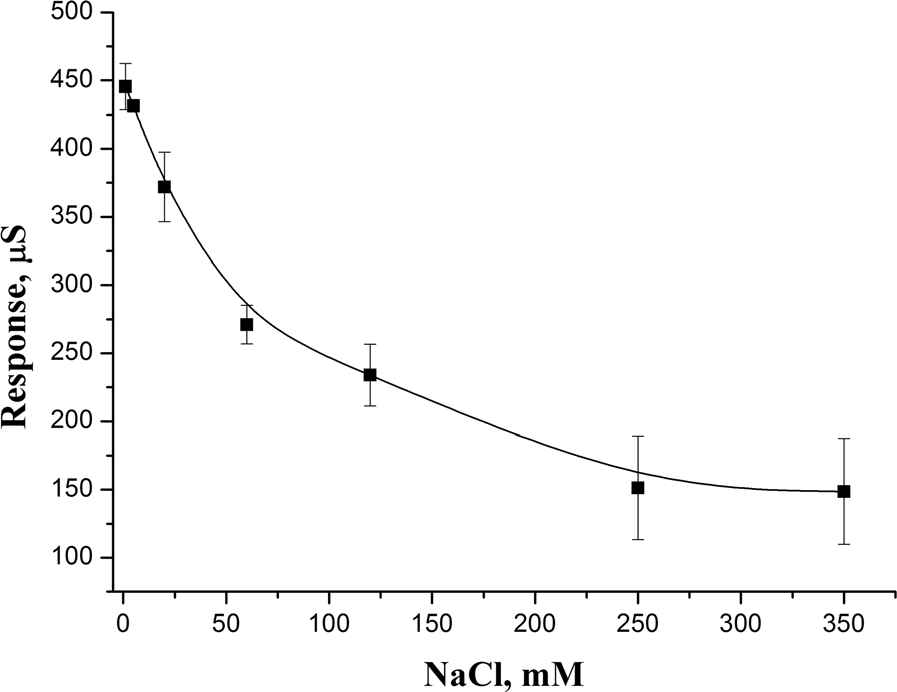
Dependence of value of biosensor response to urea concentration on various concentrations of NaCl. Measurements were carried out in 10 mM phosphate buffer, pH 7.35

One of the important characteristics of the buffer solution, which may have a negative effect on the function of conductometric biosensor is ionic strength. The main salt component of blood is sodium chloride, with a concentration of 150 mM. To study this negative effect, the signals to the same substrate concentration were measured adding NaCl of different concentrations (from 1 to 350 mM) to the solution (Fig. 4). As seen, an increase of ionic strength caused exponential decrease of the response to substrate. At NaCl concentration of 350 mM, the signal value was 34 % of the initial response to urea (in the cell without NaCl). One of the main reasons of this effect is an increase in the solution background conductivity, which at the same time enhances the noise. This can be clearly seen from an increase in the standard deviation at higher salt concentrations.

**Fig. 5:**
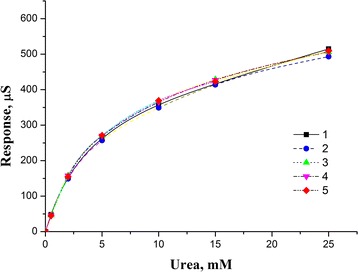
Dependence of values of biosensor responses to urea concentration on various protein concentrations in solution (*1* 0 % BSA, *2* 0.1 % BSA, *3* 0.25 % BSA, *4* 0.5 % BSA, *5* 1 % BSA). Measurements were carried out in 10 mM phosphate buffer, pH 7.35

Considering the possible non-specific binding of the enzyme and proteins in blood, which may influence the specific analysis, we used BSA to check this effect. The dependence of biosensor responses on the urea concentration at various protein concentrations solution is shown in Fig. 5. As seen, an increase of protein concentrations have not affected the response to substrate. It also confirms that the developed biosensor can be successfully used for the urea analysis in real biological samples.

### Operational Stability and Response Reproducibility of Biosensor

**Fig. 6:**
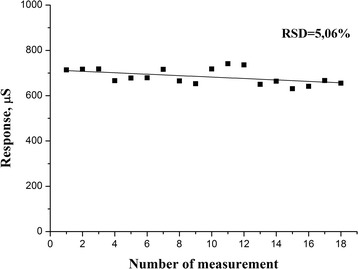
Signal reproducibility of biosensor based on recombinant urease over one working day. Measurements were carried out in 10 mM phosphate buffer, pH 7.35

Reproducibility and operational stability are the most important characteristics of biosensors. To determine the reproducibility of biosensor, the responses to the same urea concentration (12 mM) were measured over one working day with 30-min intervals, the biosensor being remained between measurements in the working buffer with constant stirring. The relative standard deviation was 5.06 %, which is quite acceptable; therefore, theoretically, the biosensor can be used to determine urea in biological samples (Fig. 6).

**Fig. 7:**
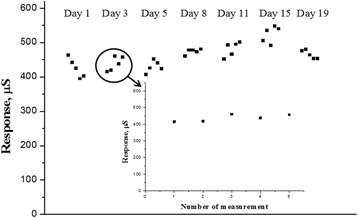
Signal reproducibility of biosensor based on recombinant urease over 19 days. Measurements were carried out in 10 mM phosphate buffer, pH 7.35

A common shortcoming of biosensors with adsorbed enzymes is the fact that enzymes are gradually washed off with the working solution because of a weak link between the enzyme and adsorbent. Therefore, it was important to check the stability of the biosensor operation for several days. When not used, the biosensors were kept dry at room temperature. Over 19 days, the responses of biosensors have not undergone any loss of activity, which is a good indicator of operational stability (Fig. 7). The biosensors based on the recombinant urease adsorption on silicalite had better reproducibility as compared to the biosensors with the urease immobilized in glutaraldehyde vapor [30].

**Fig. 8:**
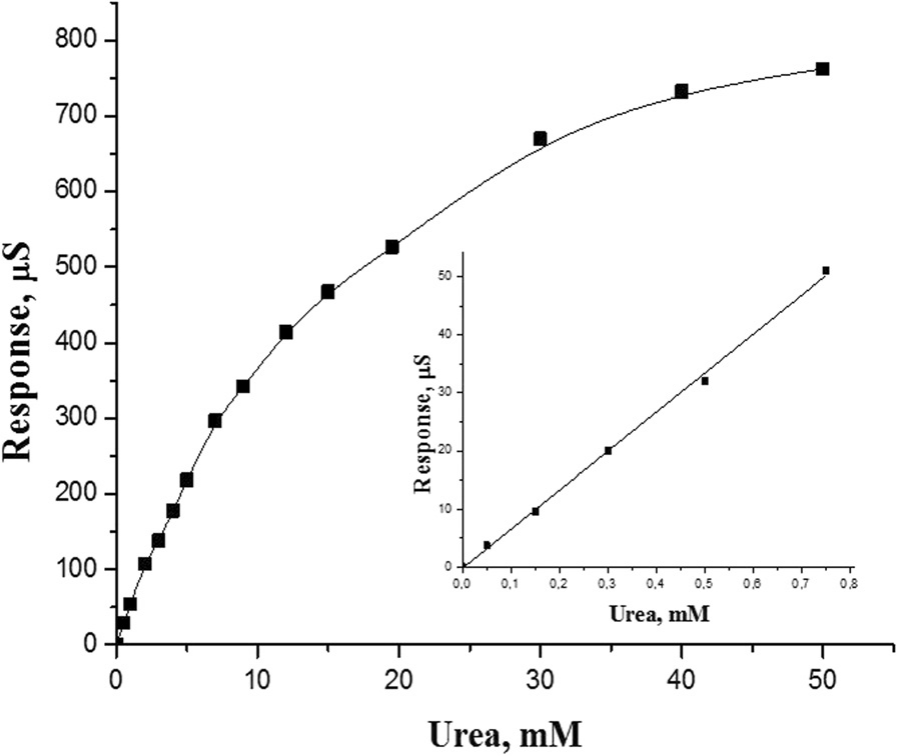
Dependence of biosensor responses to urea concentration in solution. Measurements were carried out in 10 mM phosphate buffer, pH 7.35

At the last stage of this work, a sensitivity of the biosensor based on silicalite-adsorbed recombinant urease to different urea concentrations was studied. The response dependence on the urea concentration in the analyzed sample was determined, and a calibration curve was plotted (Fig. 8). The biosensor based on recombinant urease had a wider linear range of urea determination (0.5–15 mM) and shifted toward higher concentrations (15 mM) as compared with the biosensor based on natural non-modified urease (0.025–0.75 mM) [26]. To determine the detection limit, the standard deviation of the baseline noise signal was multiplied by 3. The detection limit was 20 μM. This linear range is sufficient for the urea analysis in real biological samples.

## Conclusions

The biosensor based on recombinant urease adsorbed on silicalite was developed to determine the concentration of urea in biological samples. To adapt the biosensor to the work with real samples, its sensitivity to urea was tested depending on the concentration of the working buffer and the salt and protein concentration in the samples. The biosensor developed using the proposed method of immobilization was characterized by high operational stability over 19 days. A significant extension of the linear range of urea determination was demonstrated. This enables an analysis of the samples with high urea concentrations without significant dilution.

The developed biosensor with improved analytical characteristics may be used in biomedical and clinical diagnostics.
